# Preliminary clinical experience applying donor-derived cell-free DNA to discern rejection in pediatric liver transplant recipients

**DOI:** 10.1038/s41598-020-80845-6

**Published:** 2021-01-13

**Authors:** Dong Zhao, Tao Zhou, Yi Luo, Cheng Wu, Dongwei Xu, Chengpeng Zhong, Wenming Cong, Qiang Liu, Jianjun Zhang, Qiang Xia

**Affiliations:** 1 Department of Liver Surgery, Renji Hospital, School of Medicine, Shanghai Jiao Tong University, No. 1630 Dongfang Road, Shanghai, 200127 People’s Republic of China; 2 Department of Health Statistics, Second Military Medical University, Shanghai, People’s Republic of China; 3 Department of Pathology, Eastern Hepatobiliary Surgical Hospital, Second Military Medical University, Shanghai, People’s Republic of China; 4 Department of Pathology, Renji Hospital, School of Medicine, Shanghai Jiao Tong University, Shanghai, People’s Republic of China

**Keywords:** Diagnostic markers, Liver

## Abstract

Donor-derived cell-free DNA (dd-cfDNA) has been of major interest recently as a non-invasive marker of graft injury, but has not yet been extensively tested in children. From May to September in 2019, a total of 76 pediatric patients receiving a liver graft were enrolled and there were 27 patients excluded. Ultimately plasma samples and matched liver specimens from 49 patients were successfully collected whenever rejection was suspected clinically. Dd-cfDNA were analyzed and then compared to biopsy. Of these, 11 (22.4%) patients were found to have rejection by biopsy. Dd-cfDNA levels were higher among patients with rejection compared to those with no rejection. In subgroup analysis, dd-cfDNA% among patients with rejection differed from those with EBV/CMV infection and DILI patients. Similarly, observations were available concerning dd-cfDNA (cp/mL). The AUC for dd-cfDNA% and dd-cfDNA (cp/mL) were 0.878, 0.841, respectively, both of which were higher than conventional LFTs. For rejection, dd-cfDNA% ≥ 28.7% yielded a sensitivity of 72.7%, specificity 94.7% and dd-cfDNA (cp/mL) ≥ 2076 cp/mL, yielded a sensitivity of 81.8%, specificity 81.9%. Of note, the dd-cfDNA distribution was significantly different between whole liver and LLS transplantation. In the setting of pediatric LTx, dd-cfDNA appears to be a sensitive biomarker indicating the presence of rejection.

International Clinical Trails Registry Platform: ChiCTR1900022406.

## Introduction

Liver transplantation (LTx) is an effective therapeutic approach in the management of patients who have significant complications due to end-stage liver diseases (ESLD). However, graft injuries, including ischemia/reperfusion, infection or acute rejection, have a direct effect on survival following LTx. Expeditious detection of rejection is vital to ensure optimal long-term outcomes. At present, liver function tests (LFTs) are useful in the evaluation of graft function, but not sufficient to assess antibody-mediated rejection or acute cellular rejection after LTx. Therefore, biopsy is frequently requested to prompt further accurate clinical assessment. Apart from the potential risks and inconvenience, repeatedly serial biopsies are costly, sampling-error prone, and also have restricted specificity and sensitivity limited by subjective interpretation.

Numerous attempts have focused on developing non-invasive biomarkers for the sake of minimizing the need for performing liver biopsies and achieving more precise diagnosis of graft dysfunction. Several potential biomarkers, either biliary or blood markers, have been described. Unfortunately, none of these showed superior specificity and sensitivity over histological examination^1,2^. Cell-free DNA (cfDNA) has been of major interest recently as a non-invasive marker^3,4^. The molecules are mainly short fragments which are detectable in both the urine and blood of transplant patients^5,6^. In addition, donor derived cell-free DNA (dd-cfDNA) can be measured by either qPCR (real-time) or next-generation sequencing^7–9^, and expressed either as absolute quantification in copies/milliliter (cp/mL) or dd-cfDNA percentage (dd-cfDNA/total cfDNA). One of the earliest studies defining the role of graft-derived cell-free DNA (gcfDNA) demonstrated the presence of donor DNA in patient circulation as a useful marker of graft rejection^5^. More recently, cfDNA has also been proposed as a non-invasive biomarker specifying allograft rejection or injury in LTx. Schütz et al.^10^ revealed that the plasma mean dd-cfDNA can hardly be detected in cytomegalovirus (CMV) positive LT recipients despite that the amount could be temporarily elevated on day one post transplantation. Simultaneously, only slightly higher values were observed from rejection-free and hepatitis C virus positive patients. Beck et al. showed that dd-cfDNA fraction remained at a relative low level 10 days post-transplantation in well recovery LT patients whereas in recipients with rejection it eventually increased to 55–60%^7^.

Up to date, there are very limited reports defining the usefulness of serum liver cfDNA quantification in pediatric patients. The aim of this study was to specify dd-cfDNA levels in pediatric recipients with the background of different allograft injuries. Therefore, we performed a prospective study in which dd-cfDNA was quantified in plasma samples.

## Results

### Demographic, baseline characteristics and histopathologic diagnosis of patients

**Figure 1:**
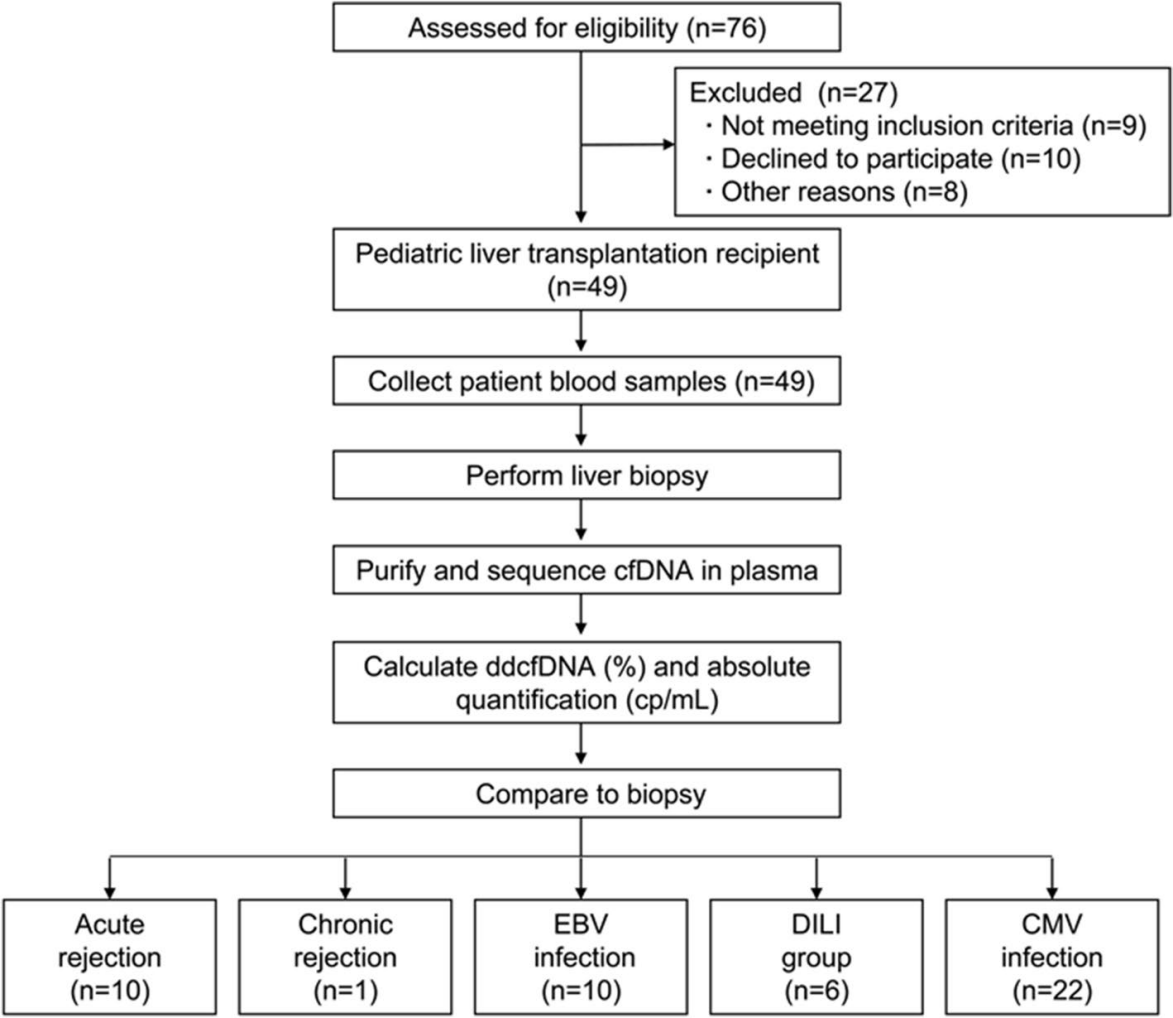
Enrollment of patients, collection of clinical samples, analysis workflow and subgroups information. Pediatric recipients with ESLD listed for liver transplantation at Renji Hospital were enrolled. Multi-organ transplant recipients were excluded. From May to September in 2019, a total of 76 pediatric patients receiving a liver graft were enrolled and there were 27 patients excluded as described in Suppl Table 1. Ultimately plasma samples and matched liver specimens from 49 patients were successfully studied. All the samples were collected whenever rejection was suspected clinically. In each case, blood samples were collected 2 h before biopsy procedures being performed. Data on the fraction of dd-cfDNA (dd-cfDNA%) and absolute number of dd-cfDNA copies per mL of plasma were compared to liver biopsy results.

A prospective diagnostic study was carried out to evaluate the performance of dd-cfDNA for the monitoring of graft rejection after LT. From May to September in 2019, a total of 76 pediatric patients receiving a liver graft were enrolled and there were 27 patients excluded as described in Suppl Table 1. Ultimately plasma samples and matched liver specimens from 49 patients were successfully studied (Fig. 1). Baseline characteristics of patients assessed are shown in Table 1 and donors’ information of donation after circulatory death (DCD) is shown in Suppl Table 2. Pediatric recipients accepting LTx included 17 males (34.7%) and 32 females (65.3%) with a median age of 19.4 months (range 5–132 months). Among them, 14 patients (28.6%) received whole liver LTx while 35 patients (71.4%) received left lateral segment (LLS) LTx. The primary indications for LTx were biliary atresia (BA) (n = 40, 81.6%), progressive familial intrahepatic cholestasis (PFIC) (n = 3, 6.1%), Wilson disease (n = 4, 8.2%) and Glycogen storages disorders (n = 2, 4.1%). The median time at which samples were collected was 7.5 months (range 3.6–12.6 months) post-operation. According to biopsy results, 11 patients (22.4%) had proven rejection (ten had acute cellular rejection and one chronic rejection), ten had Epstein–Barr virus (EBV) infection (20.4%), six had drug-induced liver injury (DILI) (12.3%) and 22 had CMV infection (44.9%).

**Table 1:** Demographics and baseline characteristics.

Characteristic	Overall (N, percentile or median)	Rejection (N, percentile or median)	No rejection (N, percentile or median)			p-value
			DILI	EBV	CMV	
Age (months)	19.4 (5–132)	12.7 (5–36)	23.8 (7–96)	22.8 (6–48)	20.8 (7–132)	0.587
**Gender**						0.344
Male	17 (34.7%)	2 (4.1%)	2 (4.1%)	3 (6.1%)	10 (20.4%)	
Female	32 (65.3%)	9 (18.3%)	4 (8.2%)	7 (14.3%)	12 (24.5%)	
**Indications for LTx**						0.384
Biliary atresia	40 (81.6%)	10 (20.4%)	3 (6.1%)	10 (20.4%)	17 (34.7%)	
PFIC	3 (6.1%)		1 (2%)		2 (4.1%)	
Wilson disease	4 (8.2%)		2 (4.1%)		2 (4.1%)	
Glycogen storages disorders	2 (4.1%)	1 (2.05%)			1 (2.05%)	
Mons post-LTx	7.5 (3.6–12.63)	2.5 (1.8–12.25)	4.6 (0.55–20.88)	11.5 (7–12.5)	6.5 (2.63–21.25)	0.971
**Surgical procedures**						0.066
Whole liver	14 (28.6%)	7 (14.3%)	2 (4.1%)	2 (4.1%)	3 (6.1%)	
LLS	35 (71.4%)	4 (8.2%)	4 (8.2%)	8 (16.3%)	19 (38.7%)	

*DILI* drug-induced liver injury, *EBV* Epstein–Barr virus, *CMV* cytomegalovirus, *PFIC* progressive familial intrahepatic cholestasis, *LLS* left lateral segment.

### dd-cfDNA levels in rejection and non-rejection group

**Figure 2:**
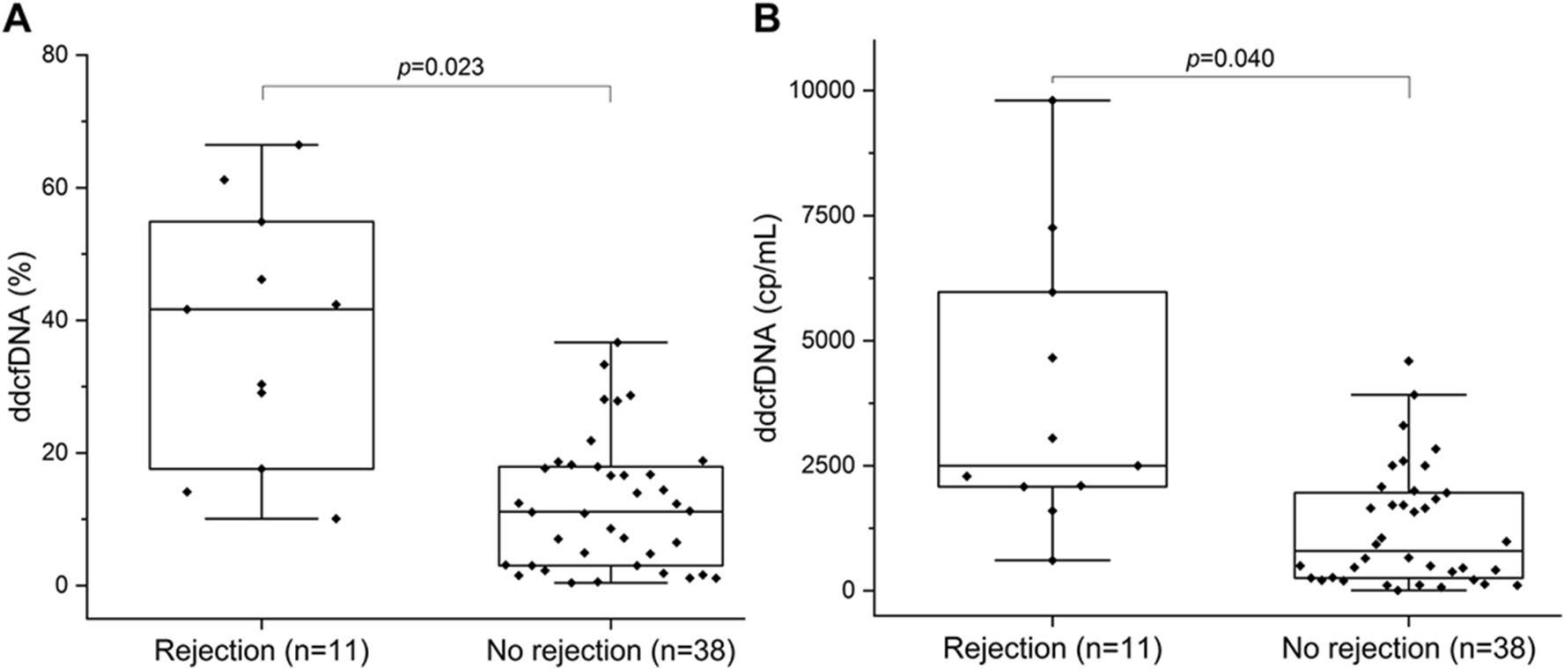
dd-cfDNA levels in rejection and no rejection group. (**A**) Box plots of plasma dd-cfDNA%, horizontal line represents the median; bottom and top of each box represents 25th and 75th percentiles. Dots are individual values. dd-cfDNA fraction (%) in rejection and no rejection group, p values were determined by Mann–Whitney U test. (**B**) dd-cfDNA (cp/mL) in Rejection and no rejection group.

We first examined the distribution of dd-cfDNA results in the rejection and non-rejection group. In terms of dd-cfDNA fraction (%), there was significant difference between the rejection (n = 11, median 41.7%, IQR 17.6–54.9%) and no rejection groups (n = 38, median 11.2%, IQR 3.0–18.0%) (p = 0.023) (Fig. 2A). The values of dd-cfDNA (cp/mL) were also statistically different among those with rejection (median 2500 cp/mL, IQR 2081–5972 cp/mL) and those with no rejection (median 796 cp/mL, IQR 245–1970 cp/mL) (p = 0.040) (Fig. 2B). In addition, ccorrelation analysis of dd-cfDNA (%) and dd-cfDNA (cp/mL) were performed and no obvious discrepancy was observed between them, therefore, the accuracy of data was confirmed (r = 0.723, p < 0.001) (Fig. S1).

### dd-cfDNA levels in different subgroups

**Figure 3:**
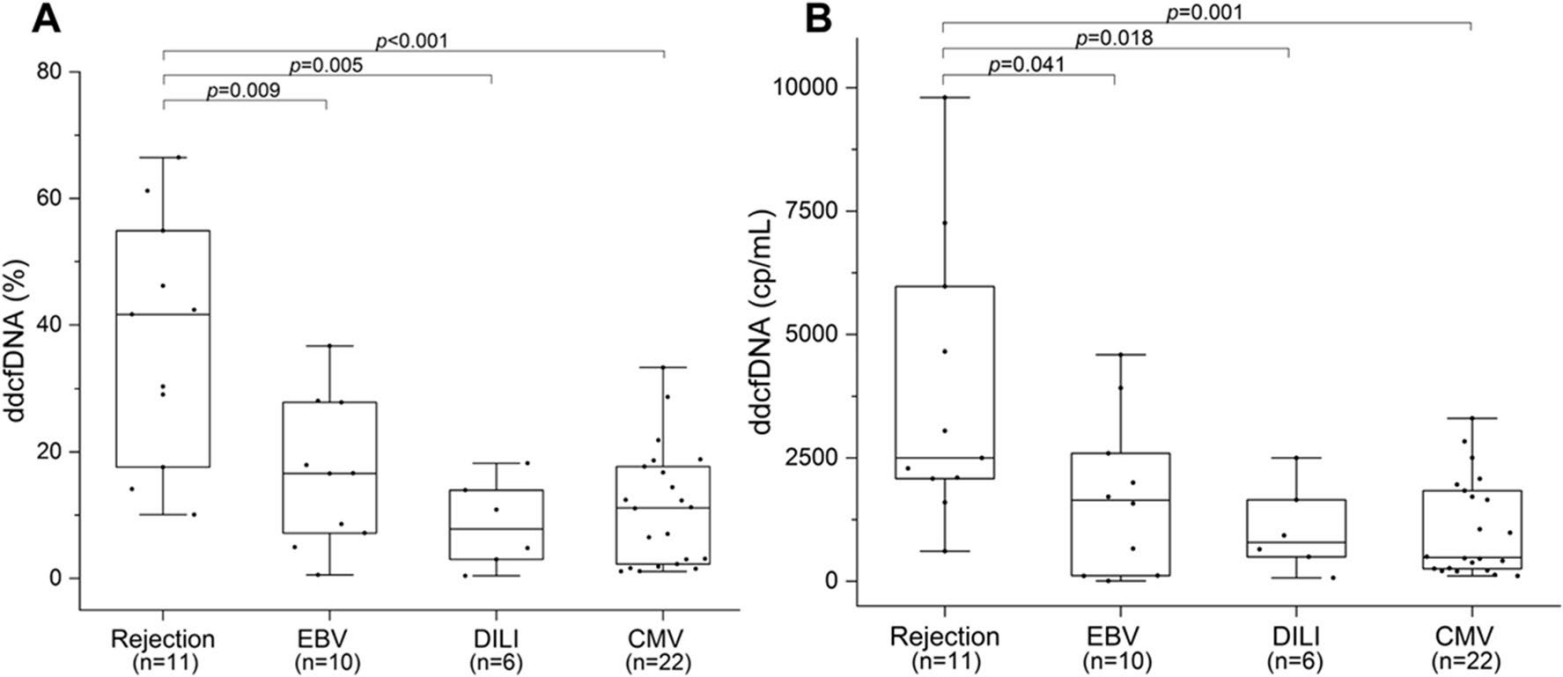
dd-cfDNA levels in different subgroups. (**A**) Box plots of plasma dd-cfDNA%, horizontal line represents the median; bottom and top of each box represents 25th and 75th percentiles. Dots are individual results. (**B**) Box plots of plasma dd-cfDNA (cp/mL). p-values were determined by Mann–Whitney U test.

Furthermore, Fig. 3 summarizes dd-cfDNA fraction (%) and absolute quantification obtained from DILI and viral infection (EBV/CMV patients) subgroups. Regarding the dd-cfDNA fraction (%), results showed no significant differences among these three subgroups. In contrast, the rejection group, with the median 41.7% (IQR 17.6–54.9%), had higher levels than other subgroups. It was significantly different in EBV infection (median 16.6%, IQR 6.61–27.9%, p = 0.009), DILI (median 7.8%, IQR 2.4–15.0%, p = 0.005) and CMV infection group (median 11.2%, IQR 2.2–17.9%, p < 0.001) (Fig. 3A).

For absolute quantification, the median dd-cfDNA (cp/mL) in rejection, EBV infection, DILI, CMV infection group were 2500 cp/mL (IQR 2081–5972 cp/mL), 1643 cp/mL (IQR 113–2925 cp/mL), 789 cp/mL (IQR 390–1863 cp/mL), and 482 cp/mL (IQR 245–1867 cp/mL), respectively. Those with rejection had higher dd-cfDNA levels than the EBV infection (p = 0.041), DILI (p = 0.018) and CMV infection subgroups (p = 0.001) (Fig. 3B). Consistent with dd-cfDNA (%) results, there were no significant differences in dd-cfDNA (cp/mL) in DILI, EBV/CMV infection groups (p > 0.05) (Table 2).

**Table 2:** Summary of statistical significances (*p*-values) from different subgroup.

Group	dd-cfDNA (%)			dd-cfDNA (cp/mL)		
	EBV+	DILI	CMV	EBV+	DILI	CMV
Rejection	0.009*	0.005*	< 0.001*	0.041*	0.018*	0.001*
EBV+		0.159	0.272		0.448	0.555
DILI			0.576			0.889

Bold and “*” represent significant differences (p-values < 0.05).

*DILI* drug-induced liver injury, *EBV* Epstein–Barr virus, *CMV* cytomegalovirus, *dd-cfDNA* donor-derived cell-free DNA.

### Diagnostic performance

**Figure 4:**
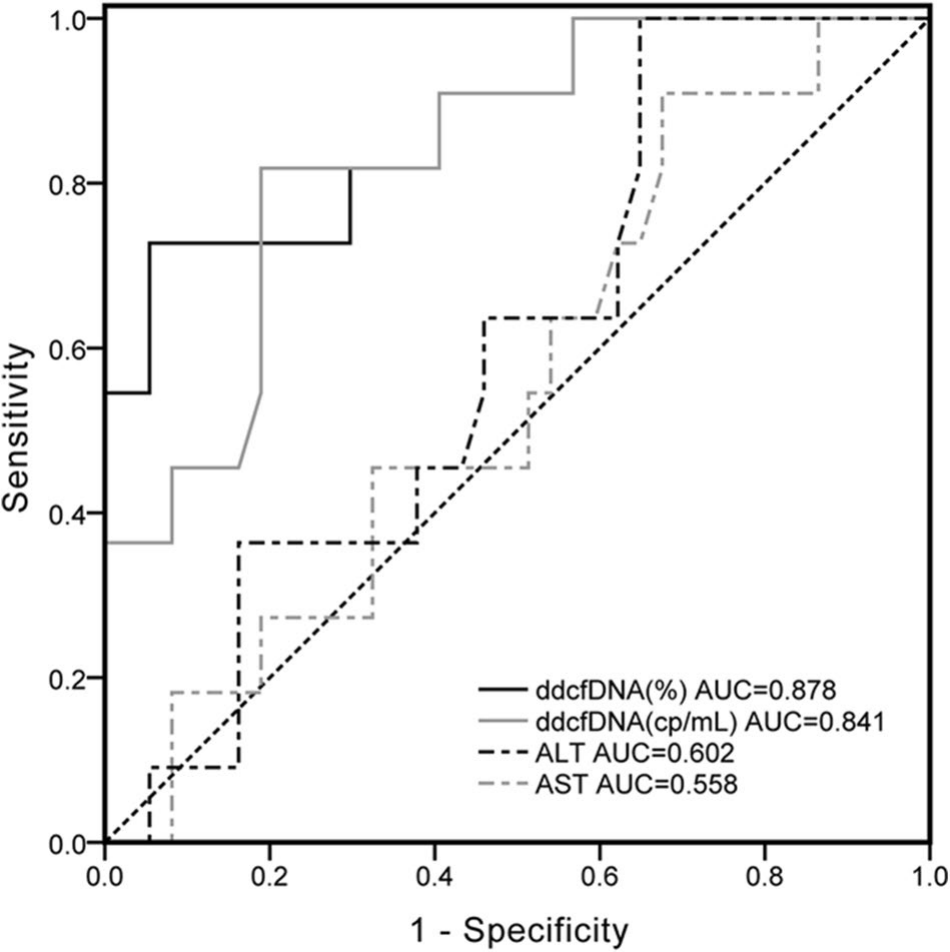
dd-cfDNA as a marker for pediatric liver transplant rejection. Black dotted diagonal represents the reference line. Black solid line represents the dd-cfDNA fraction (%) curve. Gray solid line represents the absolute quantitative (cp/mL) curve. Black dashed line represents ALT curve while gray dashed line represents AST curve.

To compare the diagnostic performance of the conventional clinical biomarker (AST/ALT) to our new measurement methods (dd-cfDNA fraction [%] and dd-cfDNA [cp/mL]), ROC curves were plotted and the area under the curve (AUC) was calculated. Compared to traditional LFTs (AST, ALT), there was much better discrimination in dd-cfDNA results between patients with rejection and no rejection (including EBV, DILI and CMV patients). Figure 4 shows the ROC curve for the outcomes of rejection diagnosis. For the dd-cfDNA, the AUC of dd-cfDNA (%) was better, whereas dd-cfDNA (cp/mL) had the weaker separation. The area under the ROC curve (dd-cfDNA (%)) was 0.878 (95% CI 75.3–95.4%). The optimal cut-point was a dd-cfDNA (%) threshold of 28.7%, which was associated with a sensitivity of 72.7% (95% CI 39–94%) and specificity of 94.7% (95% CI 82.3–99.4%). The associated PPV and NPV was 80% (95% CI 49.7–94.2%) and 92.3% (95% CI 82–96.9%), respectively. Regarding dd-cfDNA (cp/mL), the AUC was 0.841 (95% CI 70.8–93%) and optimal cut-point for a positive test result was 2076 cp/mL, yielding a sensitivity of 81.8% (95% CI 48.2–97.7%), specificity of 81.9% (95% CI 65.7–92.3%), PPV of 56.2% (95% CI 38.4–72.6%), and NPV of 93.9% (95% CI 81.4–98.2%). ROC analyses of the performance of traditional LFTs (ALT, AST) in distinguishing rejection from no rejection yielded an AUC of 0.602 for ALT and 0.558 for AST (Table 3).

**Table 3:** Diagnostic performance from ROC (%) in rejection *vs.* no rejection group.

Biomarker	AUC	Sensitivity	Specificity	PPV	NPV	Cutoff
ddcfDNA (%)	0.878 (95 CI 75.3–95.4)	72.7 (95 CI 39–94)	94.7 (95 CI 82.3–99.4)	80.0 (95 CI 49.7–94.2)	92.3 (95 CI 82.0–96.9)	28.7%
ddcfDNA (cp/mL)	0.841 (95 CI 70.8–93.0)	81.8 (95 CI 48.2–97.7)	81.9 (95 CI 65.7–92.3)	56.2 (95 CI 38.4–72.6)	93.9 (95 CI 81.4–98.2)	2076 cp/mL
ALT	0.602 (95 CI 45.1–74.0)	100 (95 CI 71.5–100)	35.14 (95 CI 20.2–52.5)	31.4 (95 CI 26.6–36.7)	100	258 U/L
AST	0.558 (95 CI 40.7–70.1)	90.9 (95 CI 58.7–99.8)	32.43 (95 CI 18.0–49.8)	28.6 (95 CI 23.0–34.9)	92.3 (95 CI 63.6–98.8)	224 U/L

*AUC* area under the curve, *dd-cfDNA* donor-derived cell-free DNA, *ALT* alanine aminotransferase, *AST* aspartate aminotransferase, *95% CI* 95% confidence interval, *PPV* positive predictive value, *NPV* negative predictive value.

The dd-cfDNA showed a higher discrimination ability than LFTs in discriminating rejection from the different subgroups of liver injury (Rejection vs. EBV, Rejection vs. DILI, Rejection vs. CMV). In terms of EBV, CMV and DILI, only a weak discrimination ability was shown. The diagnostic performance of dd-cfDNA in different subgroups is summarized in Table 4.

**Table 4:** Comparative analysis of different subgroups of AUC values.

Group	dd-cfDNA (%)			dd-cfDNA (cp/mL)			ALT			AST		
	EBV	DILI	CMV	EBV	DILI	CMV	EBV	DILI	CMV	EBV	DILI	CMV
Rejection	0.836	0.924	0.879	0.764	0.856	0.866	0.595	0.515	0.63	0.664	0.348	0.567
EBV		0.717	0.614		0.617	0.56		0.422	0.576		0.217	0.424
DILI			0.421			0.514			0.641			0.71

*DILI* drug-induced liver injury, *EBV* Epstein–Barr virus, *CMV* cytomegalovirus, *dd-cfDNA* donor-derived cell-free DNA, *ALT* alanine aminotransferase, *AST* aspartate aminotransferase.

### dd-cfDNA levels in different donation mode

**Figure 5:**
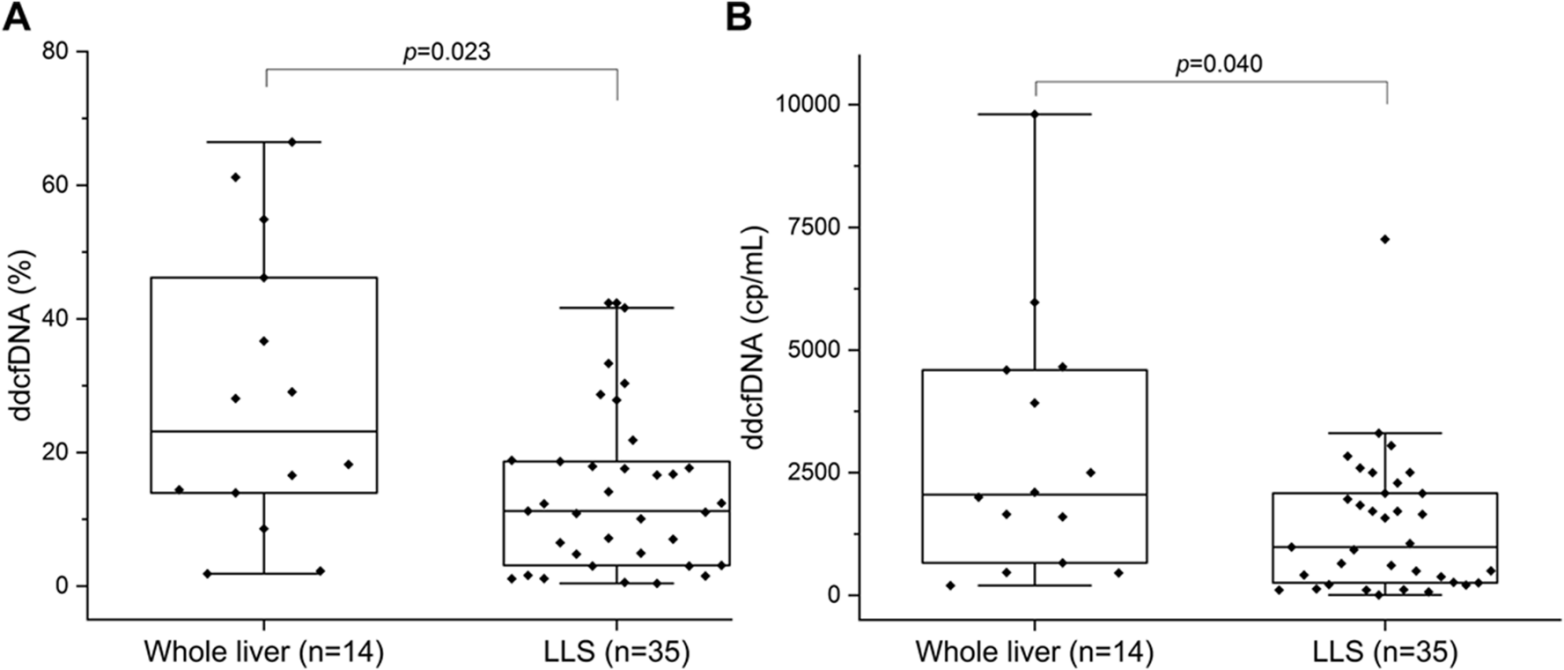
dd-cfDNA levels in different surgical procedures. (**A**) Box plots of plasma dd-cfDNA% in different operation types, horizontal line represents the median; bottom and top of each box represents 25th and 75th percentiles, Dots are individual values. (**B**) Box plots of plasma dd-cfDNA (cp/mL) in different operation procedure. *LLS* left lateral segment.

At present, there are two main donation modes and surgical procedures for pediatric LTx in our study, whole liver from DCD and LLS from living donor. The dd-cfDNA levels in different procedures were also analyzed. As Fig. 5 depicts, those received whole liver from DCD had higher dd-cfDNA fraction (%) (23.1%, IQR 12.6–48.4%) than those with LLS LTs (11.2%, IQR 3.1–18.6%) (p = 0.023). In terms of dd-cfDNA (cp/mL), the whole liver LTx median was 2051 cp/mL (IQR 614–4610 cp/mL), twofold higher than LLS LT (median 984 cp/mL, IQR 254–2081 cp/mL) (p = 0.040). A further subgroup analysis was performed to compare the dd-cfDNA in the rejection and no rejection patients within the LLS group and the whole liver group. As Supplementary Fig. S2 shows, among those received whole liver from DCD, patients developed rejection had higher dd-cfDNA fraction (%) (46.2%, IQR 28.1–61.2%) and dd-cfDNA (cp/mL) (3919.9, IQR 2000–5972) compared to those with no rejection (dd-cfDNA%, 14%, IQR 2.3–18.2%, p = 0.007; dd-cfDNA, 663 cp/mL, IQR 458.2–2500, p = 0.038) (Fig. S2A,B). Similarly, dd-cfDNA fraction (%) and dd-cfDNA (cp/mL) were higher in patients who had rejection compared to those with no rejection among those received LLS (36% vs 10.9%, p = 0.01; 2669.4 cp/mL vs 649.3 cp/mL, p = 0.005) (Fig. S2C,D).

## Discussion

Timely detection and treatment of rejection is critical to optimize LT outcomes. Currently, the common means to assess allograft function is to take liver biopsy. However, liver biopsy comes with risk and it is inconvenient, costly, and often with low-yield of tissue, sampling-error prone. Its limitations also include subjective interpretation of the results and difficulty in maneuver due to poor-compliance in children. Given these limitations of biopsies, there is an urgent need to develop noninvasive biomarkers to monitor allograft quality and function. In the present prospective study, we focused on pediatric recipients, and applied both dd-cfDNA fraction (%) and its absolute quantification to LT. The level of dd-cfDNA in patients with rejection was statistically different from those with non-rejection. The dd-cfDNA% was elevated among patients with rejection compared to those with EBV infection, DILI and CMV patients. Significant differences of dd-cfDNA distribution were observed in recipients who received different type of liver grafts: whole graft vs LLS. We have shown that dd-cfDNA is more reliable to discriminate rejection from no rejection.

Organ transplants are also genome transplants^9^, a fact that enables monitoring for allograft injury through detection of cfdDNA in the recipient’s plasma^11,12^. However, dd‐cfDNA has not been available until very recently, therefore lack of hand-on experience using it for detection of rejection in liver, especially in pediatric liver, is still plaguing us. Although the validity of cfDNA in measuring acute rejection after liver transplantation has been tested^10^, several defects existed. The GcfDNA percentage is a reflection of the relative percentage of graft cfDNA in plasma. Thus, any change in recipient or graft cfDNA can affect it. Leukocytosis as well as leukopenia might also alter the GcfDNA percentage^13^. In view of the effect of body mass index (BMI) on DNA%^14^, it is possible that the dd-cfDNA fraction (%) was related to the relative child sizes. Therefore, the dd-cfDNA fraction (%) alone is not reliable in diagnosing rejection. Here, dd-cfDNA absolute quantification was introduced and the overall superiority of dd-cfDNA was ensured by the two parameters, while the accuracy of both had also been shown by correlation analysis (Fig. S1).

Plasma dd-cfDNA fractions may reach 90% of total cfDNA immediately after liver transplantation and dd-cfDNA levels decreases to < 15% at day 10. High serum dd-cfDNA level, which dropped to a stable baseline level within several days, was found in LTx recipients very shortly after reperfusion. The peak levels of dd-cfDNA may be attributed to ischemia–reperfusion injury^15,16^. If this is the case, higher levels of dd-cfDNA in recipients with grafts from deceased donors are reasonable. Residual blood cells might be an alternative source of dd-cfDNA. Plasma dd-cfDNA fraction at day 10 post-transplantation in stable recipients with normal liver function was between 5 and 10% whereas in case of rejection it remained approximately 20% and gradually increased to 55–60%^7^. In the current study, a percentage of dd-cfDNA ≧ 28.7%, with an AUC of 0.878 (95% CI 75.3–95.4%) and yielding a 80% PPV and 92.3% NPV, could be used to discriminate biopsy-confirmed rejection. In terms of absolute quantification when dd-cfDNA ≧ 2076 cp/mL, diagnostic ability was weaker with an AUC of 0.841(95% CI 70.8–93%), yielding a 56.2% PPV and 93.9% NPV. Even though, both of them demonstrated a higher specificity compared to conventional LFTs. In addition, the dd-cfDNA levels were similar among EBV infection, DILI and CMV infection, suggesting that its level is of value for detecting rejection among those with graft injuries. However, it should be noted that the characteristics of dd-cfDNA was derived from a relatively small number of cases, therefore, its application on detecting rejection among patients with possible graft injuries should be further evaluated in a larger cohort of patients.

Children requiring lifelong IS are at high risk of developing various opportunistic infections, such as EBV/CMV. On the other hand, DILI is a health-threatening issue that may cause graft dysfunction. In our current study, DILI was triggered by either tacrolimus or Chinese herbal medicine. Traditionally, DILI has been more difficult to diagnose among patients with a history of LT given the presence of complications such as acute viral hepatitis, reperfusion injury, or acute cellular rejection^17^. Furthermore, hepatic allograft histopathological manifestations of DILI generally mimic the native livers. It is extremely difficult to distinguish hepatic-based adverse drug events from rejection. Therefore, it is essential to exclude DILI in patients with allograft dysfunction. According to our subgroup analysis, the data indicate that EBV/CMV infection does not preclude the use of donor DNA as a rejection marker. In addition, the distribution of dd-cfDNA in patients with rejection and DILI showed significant difference. However, we are not able to conclude whether dd-cfDNA could be used to discriminate viral infection and DILI from rejection, despite that dd-cfDNA values were indeed higher in rejection group than those in EBV/CMV infection and DILI group. Based on our current observation, it is impossible that single non-invasive test will emerge as the catholicon for discerning every transplant liver issue with high specificity and sensitivity. A more likely action is a blood test providing comprehensive information about alloimmune responses and cellular injury, accompanied by various clinical investigations,including aspartate transaminase, immunosuppressive drug monitoring, immunological monitoring (measurement of donor-specific antibodies), and microbial screening. According to the current data, plasma dd-cfDNA levels show marked increases both during acute rejection and graft infection, pointing to the necessity of a combined viral monitoring strategy. Although dd-cfDNA is an interesting and promising marker of solid transplant organ health, much work still needs to be done before clinical implementation.

Using dd-cfDNA as a metric, we found the difference between LLS and whole liver LTx groups (Fig. 5). It can be easily understood that the elevations of dd-cfDNA in whole liver transplantation may be more dramatic compared to that in partial liver transplantation upon graft injuries, possibly due to the close relations between the graft size and the DNA fragments originated from cell injury or death. The calculation of the dd-cfDNA fraction (%) might be a disadvantage when endeavoring to improve sensitivity. This could be attributed to possible increased the donor-derived cfDNA as well as total plasma cfDNA when compared with methods involving only absolute quantification of dd-cfDNA. From this perspective, the finding supported by definite calculation of dd-cfDNA (cp/mL) as well as dd-cfDNA fraction (%) is convincing. The results are consistent with the fact that whole liver LTx, compared to LDLT, has a high prevalence of rejection in many Asian countries^18,19^. The threshold for rejection in LDLT could be lower than that in DCD graft. This point might be independent from the surgical procedure. Therefore, surveillance after whole liver transplantation should be carefully carried out. However, according to our subgroup analysis of the LLS group and whole liver transplant group, results indicated that the dd-cfDNA levels in the rejection patients were higher than those with no rejection. The significant differences may suggest that the dd-cfDNA level might be an indication of graft injury which is from rejection, regardless of the type of transplant that the patient received.

There were several limitations in the current study. First of all, the results were constrained by sample size in a single center. Viral infection and DILI should be validated in a larger dataset. Second, the optimal dd-cfDNA threshold level for discrimination of rejection from other graft injuries was derived from one-off collected samples instead of serial ones. It would be better to obtain results from samples collected longitudinally, where the increased level of dd-cfDNA might be used to predict rejection, especially in the case of subclinical rejection when graft function is normal. Thirdly, due to the logistic reasons, we have not collected any samples from patients with normal liver function. According to Macher, higher total cfDNA and dd-cfDNA serum levels were found in patients with damage to the liver transplant (acute rejection, hepatic arterial and venous thrombosis, and profound cholestasis ending in multiple organ failure), as compared to recipients with stable graft function. In contrast, increases in total cfDNA levels but not dd-cfDNA were observed in patients with complications that did not compromise the donated organ (biliary peritonitis and surgical wound infection)^8^. Currently, the novelty and significance of these findings should be cautiously interpreted. The over-threshold level just indicates a possibility of rejection. The last but not least, dd-cfDNA was mainly evaluated on a “for-cause” basis. Carefully designed studies with normal graft function patients are needed to address higher specificity and sensitivity. Finally, there were no post-treatment values which also limits the full utility of this test.

In summary, dd-cfDNA is a promising novel biomarker which could discriminate rejection from EBV/CMV infection and DILI. It might be useful for assessing graft damages post pediatric LTx.

## Methods

### Study design and samples collection

The overall goal of this prospective study was to verify whether dd-cfDNA could be used as a marker for the detection of rejection after liver transplantation (ChiCTR1900022406). Pediatric recipients with ESLD listed for liver transplantation at Renji Hospital were enrolled. Inclusion criteria is: (1) admitted to hospital for end-stage liver diseases; (2) intended to do liver transplantation; (3) aged under 17 years male and female. Multi-organ transplant recipients were excluded. No organs from executed prisoners were used. All the samples were collected whenever rejection was suspected clinically. Blood samples were collected 2 h before biopsy procedures being performed. Data on the absolute number of dd-cfDNA copies per mL of plasma and fraction of dd-cfDNA (dd-cfDNA%) were compared to liver biopsy results. All biopsies were meticulously examined by three experienced histopathologists who were blind to the dd-cfDNA results. Rejection was diagnosed by biopsy according to Banff’s 2016 working group on liver allograft pathology. According to biopsy results, all samples were divided into rejection, EBV, DILI and CMV group. The study protocol conformed to the ethical guidelines of the 1975 Declaration of Helsinki and was approved by the ethics committee of the Renji hospital of Shanghai Jiao Tong University and a written, as well as informed consent was obtained from parents of each recipient. All researchers who performed dd-cfDNA experiments were blinded to the patients' clinical condition and biopsy results until dd-cfDNA results were finalized.

### Post-transplant therapeutic protocol

The patients were treated with standard tacrolimus based immunosuppressive regime with the target trough levels 8–12 μg/L in first 3 months. Three months post-transplant, the target trough level of 7–10 μg/L was used, and after 1 year a target trough level of 5–8 μg/L or occasionally lower was used when graft function was reliably normal.

The steroid, initially methylprednisolone intravenously administered, was converted to prednisolone at day 8 post LT. It was eventually weaned and then stopped in three months.

### DNA extraction

Blood (8 mL) was drawn into cfDNA blood collection tubes (Streck, Omaha, NE) from pediatric liver transplant recipients. gDNA was extracted from whole blood using the DNA Blood mini kit (Qiagen, Cat. No. 51104). Plasma was separated by centrifugation at 1600 × *g* for 10 min followed by a second centrifugation at 16,000 × *g* for 10 min, and was either stored at − 80 °C or immediately forwarded to cfDNA extraction using the Circulating Nucleic Acid kit (Qiagen, Cat. No. 55114).

Library construction, target region capture sequencing, bioinformatics and dd-cfDNA quantification were performed using previously published methods^20^.

### Statistical analyses

Non-parametric distributions were calculated using the Kruskal–Wallis rank sum test (multiple groups) and the Wilcoxon rank sum test (two groups). A p value < 0.05 was considered significant. Pearson's product-moment correlation and Spearman's rank correlation were used to measure relationships between different variables. A receiver operating characteristic curve (ROC) analysis was performed and the associated area under the curve (AUC) values were calculated to evaluate how well dd-cfDNA (cp/mL) and dd-cfDNA fraction (%) discriminated between rejection group and other groups. All statistical analyses were performed using R and IBM SPSS Statistics (v.19).

## Supplementary Information

Supplementary Information.

## Data Availability

The datasets generated during and/or analyzed during the current study are available from the corresponding author on reasonable request.

## Abbreviations

ALT: Alanine aminotransferase
AST: Aspartate aminotransferase
AUC: Area under the curve
BA: Biliary atresia
DILI: Drug-induced liver injury
dd-cfDNA: Donor derived cell-free DNA
DCD: Donation after circulatory death
DDLT: Deceased donor liver transplantation
EBV: Epstein–Barr virus
ESLD: End-stage liver diseases
ISD: Immunosuppressant drugs
LTx: Liver transplantation
LFTs: Liver function tests
LLS: Left lateral segment
LDLT: Living donor liver transplantation
NPV: Negative predictive value
PFIC: Progressive familial intrahepatic cholestasis
PPV: Positive predictive value
95% CI: 95% Confidence interval
