# Supplementary material for: Preliminary clinical experience applying donor-derived cell-free DNA to discern rejection in pediatric liver transplant recipients
Source: Sci Rep. 2021 Jan 13;11:1138. doi: 10.1038/s41598-020-80845-6 (PMC7807012; doi:10.1038/s41598-020-80845-6)
Supplement: Supplementary file 1 — Supplementary Information. [file 41598_2020_80845_MOESM1_ESM.pdf]

Preliminary clinical experience applying donor-derived cell-free DNA to discern rejection in pediatric liver transplant recipients

Authors: Dong Zhao, Tao Zhou, Yi Luo, Cheng Wu, Dongwei Xu, Chengpeng Zhong, Wenming Cong, Qiang Liu, Jianjun Zhang, Qiang Xia

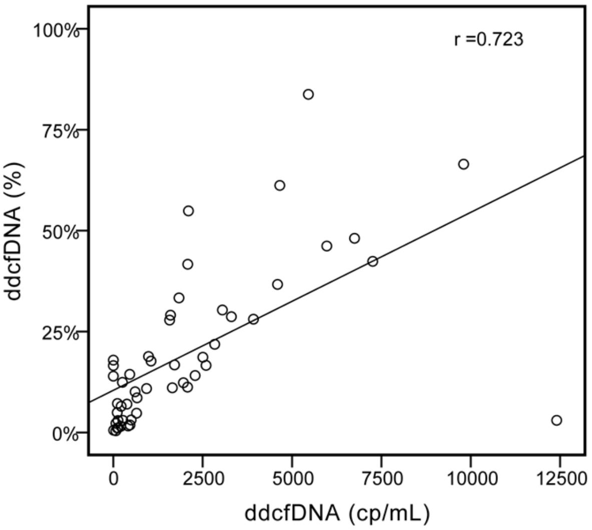

Figure S1. Correlations between dd-cfDNA (%) and dd-cfDNA (cp/ml). dd-cfDNA (%) was significantly associated with dd-cfDNA (cp/ml) with a spearman coefficient of 0.723 ($p<0.001$).

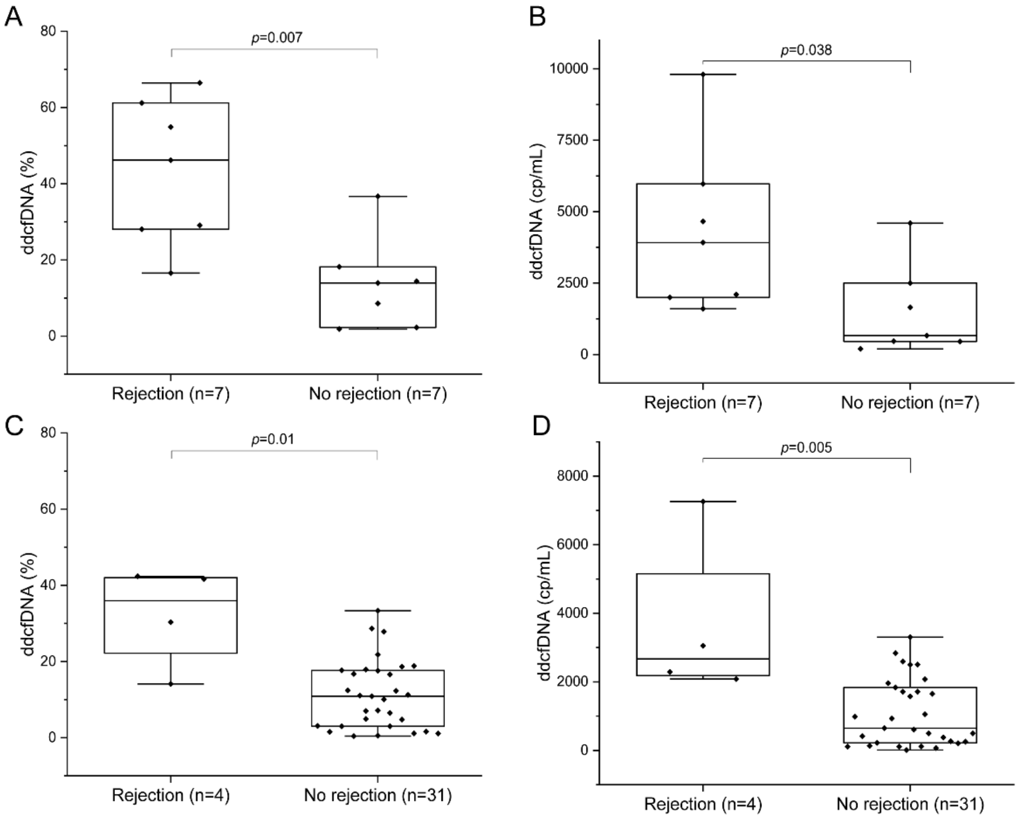

Figure S2. dd-cfDNA levels in rejection and no rejection patients within the whole liver group and the LLS group. Box plots of plasma dd-cfDNA% and plasma dd-cfDNA(cp/mL), horizontal line represents the median; bottom and top of each box represents 25th and 75th percentiles. Dots are individual values. (A) dd-cfDNA fraction (%) and (B) dd-cfDNA (cp/mL) in rejection and no rejection patients within whole liver group. (C) dd-cfDNA fraction (%) and (D) dd-cfDNA (cp/mL) in rejection and no rejection patients within LLS group. p values were determined by Mann-Whitney U test.

Suppl Table 1. Details for excluded patients

N	Reason
9	Not meeting inclusion criteria
10	Declined to participate
2	Technical failure (clotted/DNA degraded)
3	Samples not properly preserved
3	Multi-transfusion (too much foreign DNA)

Suppl Table2. Pediatric deceased donor characteristics(median/n)

**Age(mons)**	14.3
**Gender(%male)**	57.1
**Warm ischemic time(mins)**	11.3
**Cold ischemic time(hrs)**	8.2
**Causes of death(n)**	
Cerebrovascular/stroke	6
Anoxia	4
Head trauma	3
CNS tumor	1

mons, months; mins, minutes; hrs, hours; n, numbers
